# Multiplex congruence network of natural numbers

**DOI:** 10.1038/srep23714

**Published:** 2016-03-31

**Authors:** Xiao-Yong Yan, Wen-Xu Wang, Guan-Rong Chen, Ding-Hua Shi

**Affiliations:** 1 Systems Science Institute, Beijing Jiaotong University, Beijing 100044, China; 2 School of Systems Science, Beijing Normal University, Beijing 100875, China; 3 Department of Electronic Engineering, City University of Hong Kong, Hong Kong SAR, China; 4 Department of Mathematics, Shanghai University, Shanghai 200444, China; 5 Big Data Research Center, University of Electronic Science and Technology of China, Chengdu 611731, China; 6 Business School, University of Shanghai for Science and Technology, Shanghai 200093, China

## Abstract

Congruence theory has many applications in physical, social, biological and technological systems. Congruence arithmetic has been a fundamental tool for data security and computer algebra. However, much less attention was devoted to the topological features of congruence relations among natural numbers. Here, we explore the congruence relations in the setting of a multiplex network and unveil some unique and outstanding properties of the multiplex congruence network. Analytical results show that every layer therein is a sparse and heterogeneous subnetwork with a scale-free topology. Counterintuitively, every layer has an extremely strong controllability in spite of its scale-free structure that is usually difficult to control. Another amazing feature is that the controllability is robust against targeted attacks to critical nodes but vulnerable to random failures, which also differs from ordinary scale-free networks. The multi-chain structure with a small number of chain roots arising from each layer accounts for the strong controllability and the abnormal feature. The multiplex congruence network offers a graphical solution to the simultaneous congruences problem, which may have implication in cryptography based on simultaneous congruences. Our work also gains insight into the design of networks integrating advantages of both heterogeneous and homogeneous networks without inheriting their limitations.

Congruence is a fundamental concept in number theory. Two integers *a* and *r* are said to be congruent modulo a positive integer *m* if their difference *a* − *r* is integrally divisible by *m*, written as 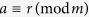
1. Congruence theory has been widely used in physics, biology, chemistry, computer science, and even music and business^2^,^3^,^4^,^5^,^6^. Because of the limited computational and storage ability of computers, congruence arithmetic is particularly useful and applicable to computing with numbers of infinite length^5^. Significant and representative applications include generating random numbers^7^, designing hash functions^8^ and checksumming in error detections^5^,^9^. As a cornerstone of modern cryptography, congruence arithmetic has been successfully used in public-key encryption^10^, secret sharing^11^, digital authentication, and many other data security applications^4^,^5^,^6^.

Despite the well-established congruence theory with a broad spectrum of applications, a comprehensive understanding of the congruence relation among natural numbers is still lacking. Our purpose is to uncover some intrinsic properties of the network consisting of natural numbers with congruence relations. A link in the congruence network is defined in terms of the congruence relation 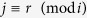, where *r* is the reminder of *j* divided by *i*. For a fixed value of *r*, we discern an infinite set of integer pairs (*i*, *j*). For each pair of such integers, a directed link from *i* to *j* (suppose *i* < *j*) characterizes the congruence relation between *i* and *j*, giving rise to a congruence network for a given reminder *r*. Let *G*(*r*, *N*) denote a congruence network, where *N* is the largest integer considered. Note that congruence networks associated with different values of *r* share the same set of nodes (integers), thereby a multiplex network^12^ with a number of layers is formed, as shown in Fig. 1(a).

To our knowledge, the multiplex congruence network (MCN) has not been explored in spite of some effort dedicated to complex networks associated with natural numbers^13^,^14^,^15^,^16^,^17^,^18^. We will demonstrate several unique and prominent properties of the MCN regarding some typical dynamical processes. Specifically, analytical results will show that all layers of the MCN are sparse with the same power-law degree distribution. A counterintuitive property of the MCN is that every layer of the MCN has an extremely strong controllability, which significantly differs from ordinary scale-free networks requiring a large fraction of driver nodes. To steer the network in a layer, the minimum number of driver nodes is nothing but the reminder *r* that is negligible as compared to the network size. The controllability of the MCN is also very robust against targeted removal of nodes but relatively vulnerable to random failures, which is also in sharp contrast to ordinary scale-free networks. This amazing robustness against attacks can be interpreted in terms of the multi-chain structure in MCN. The MCN therefore sheds light on the design of heterogeneous networks with high searching efficiency^19^ and strong controllability simultaneously. Another application of the MCN is that it can graphically solve the simultaneous congruences problem in a more intuitive way than currently used methods, such as Garner’s algorithm^20^. The solution of the simultaneous congruences problem is to locate common neighbors of relevant numbers in different layers. This alternative approach by virtue of the MCN may have implication in cryptography based on simultaneous congruences.

## Results

### Topology of MCN

**Figure 1:**
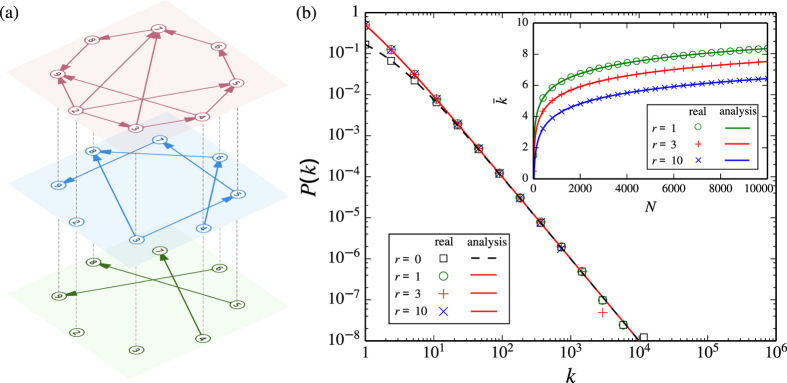
Topology of the multiplex congruence network (MCN). (**a**) An MCN with three layers *G*(*r* = {1, 2, 3}, 9), in which each direct link *L*_*ij*_ satisfies the congruence relation 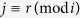. (**b**) Out-degree distributions of congruence networks with same *N* = 10000. The markers are log2-binned data. The solid lines are the analytical results from Eq. (7), and the dashed line is the analytical result from Eq. (8). The insert is the average degree 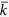 as a function of *N* for congruence networks with different *r*. The solid lines are analytical results from Eq. (9).

MCN consists of a number of congrence networks (layers) *G*(*r* > 0, *N*), as shown in Fig. 1(a). Each layer contains all the natural numbers larger than *r* but less than or equal to *N*, so the size (number of nodes) of a layer is *N* − *r*. The remainder *r* is a parameter that determines the structure of congruence network. When *r* = 0, the congruence network reduces to a divisibility network^16^, in which the dividend links to all of its divisors except itself.

The out-degrees of nodes are heterogeneous in each layer of the MCN. We have analytically derived the distribution of the out-degrees in the thermodynamic limit (see details in **Methods**):


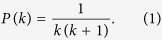


For large *k*, the out-degree distribution becomes *P*(*k*) ~ *k*^−2^, thus *G*(*r*, *N*) is a typical scale-free network. All *G*(*r* > 0, *N*) have similar out-degree distributions, as shown in Fig. 1(b), but the divisibility network *G*(0, *N*) has a different out-degree distribution. For small *k*, *P*(*k*) of *G*(0, *N*) deviates from the other networks *G*(*r* > 0, *N*). The main factor that accounts for the difference lies in that half of the nodes in *G*(0, *N*) have no outgoing links, but in *G*(*r* > 0, *N*) there are only *r* nodes without outgoing links.

Analytical results demonstrate that the average degree of any layer increases logarithmically with the network size (see Fig. 1(b) and **Methods** for details), and a larger value of *r* corresponds to a sparser layer. These results indicate that *G*(*r*, *N*) is always a sparse network. Hence, the MCN is compatible with a sparse storage, which is important for applying the MCN to solve real-world problems.

**Figure 2:**
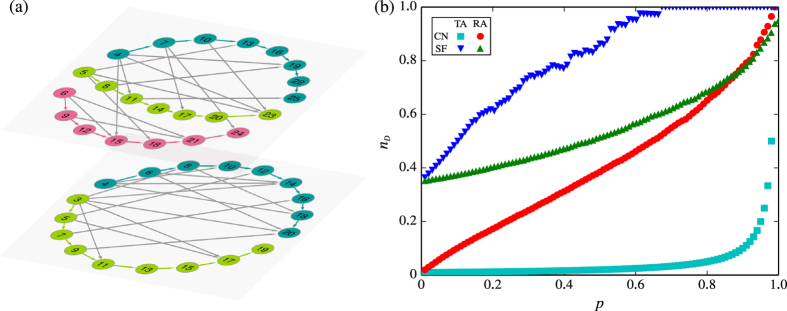
Controllability of MCN. (**a**) Multi-chain structure in MCN. Different color of nodes and links highlights different chains in two layers. In each layer, the smaller the node’s number, the larger its out-degree. (**b**) Driver node density *n*_*D*_ as a function of the proportion of removed nodes *p*. The nodes are removed according to two strategies: RA (random attacks: randomly remove *p* fraction of nodes) and TA (targeted attacks: remove the top *p* fraction of nodes according to their out-degrees). Two networks are compared: a congruence network (CN) with remainder *r* = 1 and a directed scale-free (SF) network with the same scaling exponent *γ* = 2.001 in both in- and out-degree distributions. The two networks have the same size *N* = 100 and average out-degree 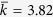.

According to the definition of MCN, the numbers in each layer *G*(*r* > 0, *N*) can be classified into *r* arithmetic sequences:


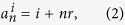


where 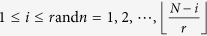 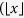 denotes the largest integer not greater than *x*). The consecutive numbers in the sequence are linked from small to large, resulting in *r* chains traversing all nodes in a layer, as shown in Fig. 2(a). The root node of a chain is the minimum number in the chain. There are totally *r* root nodes associated with *r* chains. The end of a chain is always the maximum number in the chain. Note that *r* = 0 is a special case, because the arithmetic sequence does not exist in the layer *G*(0, *N*), rendering the absence of the multi-chain structure in the divisibility network.

The above results indicate that although the divisibility network *G*(0, *N*) is a special case of *G*(*r*, *N*), it has some fundamental differences from *G*(*r* > 0, *N*). Only when *r* > 0 the multi-chain structure emerges, and the number of nodes without outgoing links is negligible. Some evidence has suggested that the multi-chain structure and the absence of nodes with low out-degrees play an important role in the controllability of complex networks^21^,^22^. Below, we will further investigate the controllability properties of the MCN.

### Controllability of MCN

In principle, the MCN composed of natural numbers is not a dynamical system, such that it cannot be controlled. However, because of the multi-chain structure, the MCN provides significant insight into the design of heterogeneous networked systems with a strong controllability. Thus, we treat the MCN as a network of dynamical systems and explore its unique and outstanding controllability properties. The central problem of controlling complex networks is to discern a minimum set of driver nodes, on which external input signals are imposed to fully control the whole network as a dynamical system. Let *N*_D_ denote the minimum number of driver nodes and *n*_D_ denote the fraction of driver nodes in a network. In general, a network with a smaller value of *n*_D_ is said to be more controllable.

According to the exact controllability theory for complex networks^23^ and the sparsity of MCN, we can prove that (see **Methods** for details)


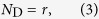


and


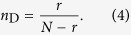


For large *N* (namely *r* ≪ *N*), *n*_D_ → 0 and *G*(*r* > 0, *N*) is considered as highly controllable. Furthermore, according to both the exact controllability theory^23^ and the structural controllability theory^24^, the driver nodes are the *r* root nodes of the chains in *G*(*r* > 0, *N*) (see details in **Methods**). Meanwhile, the driver nodes are the hub nodes with the maximum node degree. In comparison, due to the absence of chains, the divisibility network *G*(0, *N*) with 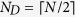 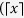 denotes the smallest integer not less than *x*) is hard to control for large *N*.

It has been recognized that scale-free networks are often difficult to control^24^. In particular, Liu *et al.*^24^ have analytically found that when the network size *N* → ∞, one must control almost all nodes in order to fully control a scale-free network with scaling index *γ* → 2. The MCN is a scale-free network with scaling exponent *γ* = 2, but one just needs to control *r* root nodes to achieve full control. Such a strong controllability stems from the inherent multi-chain structure in MCN. From the perspective of structural controllability, all nodes in the chains in MCN are matched except the *r* roots, which need to be controlled. Thus, the MCN is valuable for designing heterogeneous networks with strong controllability.

We also found that the MCN is strongly structurally controllable (SSC) because of the multi-chain structure (see **Methods**), which provides significant insight into the design of heterogeneous and controllable networks without exact link weights. A network is said to be SSC if and only if its controllability will not be affected by the link weights in its adjacency matrix^24^, or equivalently, for any distribution of link weights, the network will be fully controllable from the same set of driver nodes. The SSC property implies that the MCN is robust against the fluctuation and uncertainty of link weights. This is an outstanding feature with practical significance since sometimes link weights are hard to be exactly measured and they are sometimes time-varying in real situations.

The robustness against attacks is also a significant problem for the design of a controllable networked system^25^. We explore the robustness of the controllability of MCN against attacks to nodes and find some unique properties, which are useful for the design of practical networks. On the one hand, due to the existence of chains rooted in *r* driver nodes in MCN, targeted attacks to driver nodes will neither destroy the multi-chain structure nor increase *n*_D_. Here, nodes critical for targeted attacks can be identified based on the rank of node degrees or their hierarchical structure^26^. In MCN, driver nodes (the *r* root nodes) become such critical nodes. In this regard, MCN is robust against targeted attacks. On the other hand, random attacks to nodes may cut some chains. As a result, an additional driver is required to control each new breakpoint, leading to an increase of *n*_D_. Thus, the controllability of MCN is unusual in resisting attacks in the sense that it is robust against intentional attacks but vulnerable to random attacks, which significantly differs from general scale-free networks. The results in comparing the MCN and SF networks generated by using a static model^27^ are shown in Fig. 2(b). To make an unbiased comparison, an SF network with the same scaling exponent *γ* and 〈*k*〉 as the MCN is necessary. However, because of the graphicality constraint, it is not possible to generate a random SF network with *γ* = 2^28^,^29^. Thus, we slightly release the requirement by using a static model to generate SF networks with the same 〈*k*〉 but with *γ* = 2.001. Indeed, one can see that *n*_D_ remains nearly unchanged under targeted attacks to driver nodes; whereas random attacks to node causes clear increase of *n*_D_. This phenomenon is consistent with our analysis in terms of the multi-chain structure. Moreover, for the presence of random attacks, in a wide range of the fractions of failed nodes, the controllability of MCN is still better than SF networks in general.

### Solving the simultaneous congruences problem

One of the applications of the MCN is that one can graphically solve the simultaneous congruences problem, which has implication in communication security and computer science. In particular, one can find that MCN is exactly a topological representation of the system of simultaneous congruences^6^. A system of simultaneous congruences is a set of congruence equations:


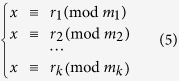


If the moduli 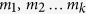 are pairwise coprime, then a unique solution modulo 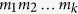 exists. This is also known as the *Chinese remainder theorem* (CRT), which has many applications in computing, coding and cryptography^4^,^5^,^6^,^30^. A well-known algorithm to solve the simultaneous congruences in CRT is the Gaussian algorithm^20^, also known as *Dayanshu*^31^ in ancient China. Here, we present an intuitive approach based on the MCN to solve the simultaneous congruences in Eq. (5). Firstly, we construct an MCN containing *k* subnetworks with remainders 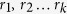, respectively. To focus on the minimum solution of Eq. (5), we set the maximum number in the network as 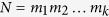. Then, we find the common successor neighbor of the nodes 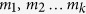 in this MCN, which is precisely the solution *x*.

We use a well-known example of CRT, recorded in *Sunzi Suanjing*^32^, to demonstrate our approach. The problem in this example is: ‘Suppose we have an unknown number of objects. When grouped in threes, 2 are left out, when grouped in fives, 3 are left out, and when grouped in sevens, 2 are left out. How many objects are there?’ This problem is equivalent to the following simultaneous congruences


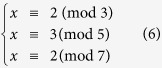


**Figure 3:**
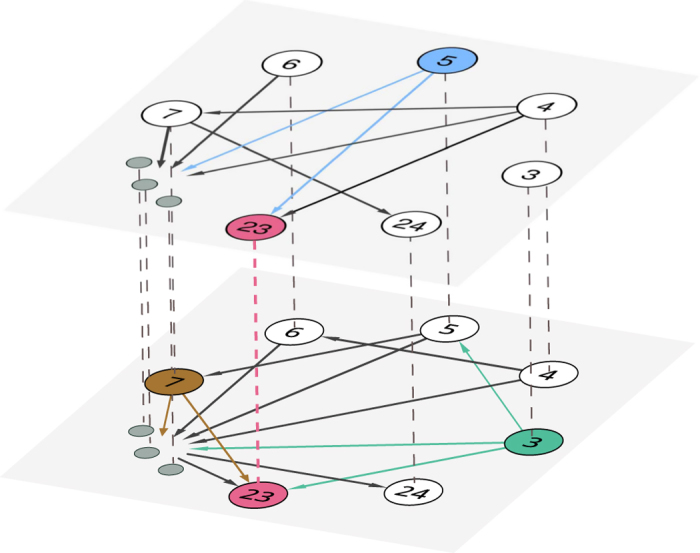
Solving simultaneous congruences using MCN. For visualization, we only show a part of nodes in the MCN. In the upper layer, the set of successor neighbors of node 5 is 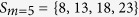, and similarly 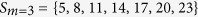 and 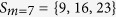 in the lower layer. Thus the common successor neighbor of the three nodes is 23, which is the solution of the simultaneous congruences problem described by in Eq. (6).

To solve the problem, we first construct an MCN of two layers, *G*(2, 105) and *G*(3, 105), as shown in Fig. 3. Then, we find the common successor neighbor of the three moduli, 3, 5 and 7, and finally get the result *x* = 23.

It is noteworthy that the traditional algorithm for solving the simultaneous congruences problem, e.g., Garner’s algorithm^20^, is more efficient than our algorithm based on the MCN that is essentially a brute-force search scheme. Thus, it is infeasible to immediately use the graphical approach in data security. However, the graphical algorithm offers a new route to the simultaneous congruences problem from the perspective of a complex network, which may be further explored for possible improvement of the currently used algorithm.

## Discussion

We have defined a multiplex congruence network composed of natural numbers and uncovered its unique topological features. Analytical results demonstrate that every layer of the multiplex network is a sparse and scale-free subnetwork with the same degree distribution. Counterintuitively, every layer with a scale-free structure has an extremely strong controllability, which significantly differs from ordinary scale-free networks. In general, a scale-free network with power-law degree distribution is harder to control than homogeneous networks. This is attributed to the presence of hub nodes, at which dilation arises according to the structural control theory^24^. As a result, downstream neighbor nodes of hubs are difficult to control. Moreover, due to a large number of nodes connecting to hubs, scale-free networks are usually of weak controllability with a large fraction of driver nodes. In contrast, in spite of the scale-free structure of the congruence network, the long chains in each layer considerably inhibit dilation and reduce the number driver nodes. Furthermore, an interesting finding is that every layer is also strong structurally controllable in that link weights have no effect on the controllability. This indicates that the controllability of the multiplex congruence network is extremely robust against the inherent limit to precisely accessing link weights in the real situation. To our knowledge, a scale-free network with strong structural controllability has not been reported prior to our congruence network.

An unusual controllability property is that the controllability of each layer is robust against targeted attacks to driver nodes, but relatively fragile to random failures of nodes, which is also different from common scale-free networks. Previously reported results^25^,^26^ demonstrate that targeted removal of high degree nodes and nodes in the top level of a hierarchical structure causes maximum damage to the network controllability. Under the two kinds of intentional removals, a network is easier to break to pieces, such that more driver nodes are required to achieve full control. Thus, targeted attacks are defined in terms of the two types of node removals. In the congruence network, high degree nodes and high level nodes are exactly identical, leading to the combination of the targeted attacks. Strikingly, the congruence network is robust against the targeted attacks, because of the existence of the chains. Targeted attacks will not destroy the chains in the downstream of the attacked node. As a result, the number of driver nodes nearly does not increase, even when a large fraction of nodes has been targeted attacked. The outstanding structural and controllability properties of the multiplex congruence network are valuable for designing heterogeneous networks with strong controllability and high searching efficiency rooted in the scale-free structure.

Another application of the multiplex congruent network is to solve the simultaneous congruences problem in a graphical and intuitive manner. The multiplex congruence network by converting the algebraic problem of solving simultaneous congruences equations to be a graphical problem of finding common neighbors in a graph, offers an alternative route to the traditional approaches. Despite this property, the traditional algorithms, such as the Gaussian algorithm and Garner’s algorithm, outperform the graphical method in computational efficiency. Hence, the graphical method is not applicable in data security at the present. Nevertheless, the graphical approach may inspire a combination of the graphical and algebraic methods to improve the current algorithms, which is potentially valuable in communication security, computer science and many fields relevant to cryptography. Our work may also stimulate further effort toward studying of networks arising from natural relationships among numbers, with outstanding features and applied values. Many topological insights can be expected from complex networks consisting of natural numbers.

## Methods

### Deriving the out-degree distribution of MCN

For a subnetwork *G*(*r* > 0, *N*) in MCN, the total number of nodes is *N* − *r* and the number of nodes without out-links is *r*. The out-degree of a node labelled *m* in the range of 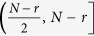 is 1, because node *m* can only link to one node i.e. node *m* + *r* in the network; similarly, the out-degree of a node labelled *m* in the range of 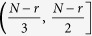 is 2, because node *m* can only link to two nodes i.e. nodes *m* + *r* and 2*m* + *r*; similar scenarios appear to the other nodes. Thus, we can derive the distribution of out-degrees in the thermodynamic limit, as follows:


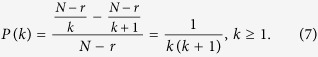


In *G*(0, *N*), the numbers larger than *N*/2 have no out-links, i.e. 
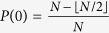
, and the numbers in the range of (*N*/3, *N*/2] have only one out-link, i.e. 
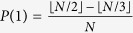
. Analogously, as *N* → ∞,


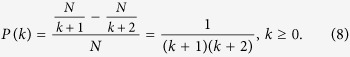


This is the same as the in-degree distribution of a growing network with copying^33^, which can be regarded as a random version of the divisibility network^16^.

### Calculating the average degree of sparse MCN

Note that the minimum number *r* + 1 in *G*(*r* > 0, *N*) has the maximum out-degree 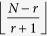 and the second minimum number *r* + 2 has the out-degree 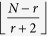, and so on. Thus, the average degree 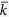 of *G*(*r* > 0, *N*) is


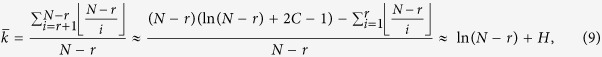


where *C* is the Euler constant and 
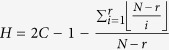
 is a constant, which is approximately *C* − 1 − ln(*r*) when *r* is very large.

Eq. (9) is not valid for the divisibility network *G*(0, *N*). In *G*(0, *N*), number 1 has the maximum out-degree *N* − 1, and 2 has the second maximum out-degree 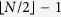, and so on. In a similar way to Eq. (9), we can obtain the average degree of *G*(0, *N*), as


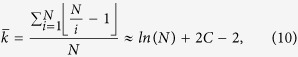


which is consistent with the analytical results provided by Shekatkar *et al.*^18^.

### Controllability of MCN and identification of driver nodes

An arbitrary network with linear time-invariant dynamics under control can be described by


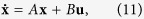


where the vector 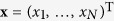 stands for the states of *N* nodes, *A* denotes the coupling matrix (transpose of the adjacency matrix) of a network, 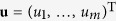 is the vector of *m* input signals, and 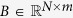 is the input matrix.

System (11) is said to be (state) controllable if the input signal **u** imposed on a minimum number *N*_D_ of driver nodes specified by control matrix *B* can steer the state **x** from any initial state to any target state in finite time. The level of controllability of the networked system (11) is defined by the fraction *n*_D_ of driver nodes in the sense that a complex network is more controllable if a smaller fraction of driver nodes is needed to achieve full control. According to Liu *et al.*^24^, the key is to find a matrix *B* associated with the minimum number of controllers to ensure full control of system (11).

Because of the sparsity of MCN, *N*_D_ of a layer *G*(*r*, *N*) is determined by^23^


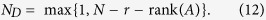


Because in *G*(*r* > 0, *N*) each node-labelled number can only link to the node-labelled numbers larger than itself, the coupling matrix *A* is a strictly lower-triangular matrix. Moreover, *A* of *G*(*r* > 0, *N*) is in a column echelon form because the minimum number linked from node *m* is always *m* + *r*. In other words, the leading coefficient of the *i*th column in *A* is precisely in the (*i* + *r*)th row. On the other hand, the last *r* columns of *A* are all zeros because the maximum *r* nodes in *G*(*r* > 0, *N*) have no out-links, so the rank of *A* is exactly *N* − 2*r*. An example of matrix *A* of *G*(1, 9) is


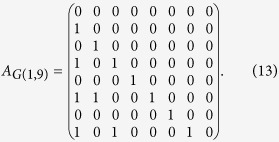


According to Eq. (12), we can show that the minimum number of driver nodes *N*_D_ for *G*(*r* > 0, *N*) is *r* and the value of non-zero elements in *A* does not affect rank(*A*), which indicate that *G*(*r* > 0, *N*) is SSC.

According to the exact controllability theory^23^, the control matrix *B* to ensure full control of the congruence network *G*(*r* > 0, *N*) should satisfy the following condition


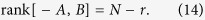


Notice that the rank of the matrix [−*A*, *B*] is contributed by the number of linearly independent rows, hence the input signals specified via *B* should be imposed on the linearly dependence rows in *A* so as to eliminate all linear correlations in Eq. (14). Apparently, the first *r* rows in the coupling matrix *A* of *G*(*r* > 0, *N*) are all zero rows (see Equation(13)), hence the *r* driver nodes that need to be controlled to maintain full control are just the minimum *r* nodes of the congruence network, i.e. the *r* roots of the chains in the congruence network (see Fig. 2(a)).

The coupling matrix of the divisibility network *G*(0, *N*) is also a strictly lower-triangular matrix and in a column echelon form, but the rank of the matrix is 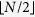, because in *G*(0, *N*) the node with labelled number larger than 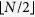 has no out-links, namely, the last 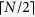 columns of the matrix are all zeros. An example of *G*(0, 9) is


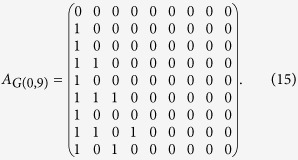


Therefore, according to Eq. (12), we finally obtain the minimum number of driver nodes of *G*(0, *N*) as 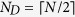, indicating that one must control half of the nodes in order to control the whole divisibility network. Moreover, the value of non-zero elements in *A* does not affect rank(*A*), which indicate that *G*(0, *N*) is SSC. Thus, the MCN composed of *G*(0, *N*) and *G*(*r* > 0, *N*) is SSC.

## Additional Information

**How to cite this article**: Yan, X.-Y. *et al.* Multiplex congruence network of natural numbers. *Sci. Rep.* **6**, 23714; doi: 10.1038/srep23714 (2016).
